# P07-05 Romanian GPs knowledge, attitudes and behavior related to physical activity on prescription

**DOI:** 10.1093/eurpub/ckac095.105

**Published:** 2022-08-29

**Authors:** Petru Sandu, Razvan Mircea Chereches, Bogdan Covaliu, Floarea Mocean

**Affiliations:** 1 Public Health, University Babes-Bolyai, Cluj-Napoca, Romania; 2 Public Health, University of Medicine and Pharmacy Iuliu Hatieganu, Cluj-Napoca, Romania

**Keywords:** physical activity on prescription, general practitioners, attitudes, behavior, developing country

## Abstract

**Background:**

Physical activity on prescription (PAP) - like schemes, have been documented to encourage sustained increases in the levels of physical activity of populations. Although proven effective, these PA promotion schemes have only been implemented high-income countries (eg. Northern/Western Europe). The aim of this study was to explore the opportunity to test PAP (related) schemes in Romania, a developing country.

**Methods:**

In the timeframe May-June 2018 we conducted a transversal study, using an online questionnaire adressed at general practitioners (GPs) in the county of Cluj. The instrument had 4 sections: 1. attitudes/opinions regarding role of GP in PA promotion; 2. GPs (current) behavior related to PA promotion in their practice; 3. Knowledge regarding the recommended PA levels for children, youth and adults; and 4. Socio-professional information.

**Results:**

A number of 84 GPs (out of 350) have completed the questionnaire, for a response rate of aproximatelly 25%, the sample being representative of the population - considering gender distribution and workplace location, urban/rural. The majority of the GPs (78%) stated that their role in PA promotion is limited to broadly disscussing this topic with their patients. The more consultations they have (less time available), the more they are prone to promote PA in their practice (contrary to current literature). Only 1 in 5 GPs have reported the correct number of minutes of PA for both adults and children & youth. The lower measured knowledge, the higher were the self-assessed levels of knowledge regarding PA benefits they report.

**Conclusion:**

Romanian GPs knowledge, attitudes and behavior related to PA prescription/recommendation are strongly influenced by lack of proper information, incentives and/or enforcement. Before attempting to introduce PAP in Romania, more education, awareness and financial/structural resources should be allocated to improve acceptability and feasibility of such PA promotion scheme.

